# A cross-sectional study of the welfare of calves raised in smallholder dairy farms in Meru, Kenya, 2017

**DOI:** 10.14202/vetworld.2018.1094-1101

**Published:** 2018-08-10

**Authors:** Emily. K. Kathambi, John A. Van Leeuwen, George K. Gitau, Shawn L. McKenna

**Affiliations:** 1 Department of Health Management, Atlantic Veterinary College, University of Prince Edward Island, Canada; 2 Department of Clinical Studies, Faculty of Veterinary Medicine, University of Nairobi, Nairobi, Kenya

**Keywords:** calf comfort, calf hygiene, dairy calves, Kenya

## Abstract

**Aim:**

This study was aimed at describing calf comfort and determining the individual and pen level factors that affect comfort status (in particular, calf leg hygiene scores) of smallholder dairy farms in Meru County, Kenya.

**Materials and Methods:**

A cross-sectional study was carried out on 52 calves that were up to 1 year old in 38 dairy farms (mean±standard deviation: Herd size=1.71±0.7 milking cows and milk production=6.7±3.1 L/day) in Meru, Kenya, in 2017, with the intention to describe their comfort and determine the factors associated with leg hygiene as a critical parameter for calf comfort assessment. Calves’ biodata, health status, and leg hygiene were assessed, along with pen characteristics such as area, hygiene, and knee impact and knee wetness scores, while a questionnaire was administered to the farmers to gather information regarding calf housing management practices in the farm.

**Results:**

The calves had a mean body weight of 85.2±32.8 kg and average daily weight gain of 0.50±0.45 kg per day. 71% of calves had a good body condition score (≥2.5), and the mean space allowance per calf was 2.52±1.56 m^2^. Approximately 75% of the calves (39/52) were kept in pens, and the rest were reared outdoors. For 39 calves kept indoors, 26% (10/39) of them had wooden or concrete floors while 74% (29/39) had dirt floors. Nearly two-thirds (62%) of indoor calves (26/39) were reared in pens with bedding, and 23% (9/39) and 33% (13/39) of the calves reared indoors were kept in pens displaying a failed knee impact test and failed knee wetness test. Indoor housed calves had an increased probability of having dirty calf legs (cleanliness score of >2.5) by 8.6 times (p=0.031), compared to outdoor-housed calves. In the final multivariable logistic regression model of 39 calves in pens, concrete or wood floors (odds ratio [OR]=7.9, p=0.047), poor body condition (OR=17.1, p=0.020) and use of bedding (OR=12.5, p=0.046) appeared to be positively correlated with dirtiness of calf legs, compared to dirt floors, good body condition, and no bedding, respectively.

**Conclusion:**

Overall, some calf comfort aspects were covered for the majority of calves examined, but 69% of the pens were categorized as dirty, especially those with wooden or concrete floors and poor bedding management. Smallholder dairy farmers in Kenya should be trained on calf housing management to improve calf comfort and productivity.

## Introduction

Animal welfare has become a global concern in livestock, companion, and wild animals [1]. To maintain body integrity while growing, calves need: Access to fresh air with sufficient oxygen, adequate resting in the right postures to prevent sleep deprivation, movement and exercise space for bone and muscle development, an adequate and well-balanced diet (colostrum, milk, and calf starter), enough water, and social contact [2]. Housing management of dairy calves can influence their lying behavior, feeding status, and social behavior with negative effects on their growth and development [2]. The recommended space allowance for young calves is 95 cm width by 135 cm length [3]. Calves kept in pens that are about 1-1.5 m in length display a higher percentage of lying behavior compared to calves kept in smaller pens [2], with factors such as pen area per calf, type of floor, type of bedding, and weather conditions playing an important role in lying behavior [2]. The housing of dairy calves can be done individually, in pairs or groups. The individual housing of calves in dairy production has raised animal welfare concerns [4,5]. Surveys in Canada and USA show that 87.9% and 65.4% of farms individually housed their calves, pre- and post-weaning, respectively [6,7]. European Union regulations indicate that group housing is mandatory for calves older than 8 weeks [8]. Benefits attributed to paired and group-housing are reduced labor requirements per calf [9], improved social behavior of calves [10,11], decreased fear for a new diet during weaning [12], higher intakes of starter feed [5], and increased weight gains [5,13-15]. The selection of individual housing is frequently based on studies showing increased weight gains [16], decreased calf morbidity [17], and less behavioral issues such as cross-sucking in single raised calves [18]. Dairy calves spend about 18 h/day lying down [13,19-21]. The lying behavior has also been used as an indicator of calves’ adaptability to new housing conditions [22]. The type of floor surface in pens influences the lying time and posture of calves [23]. Calves showed an aversion to lie down on a bare concrete versus a sawdust-bedded surface [21]. On the other hand, calves did not show any significant difference in lying behavior when kept in pens with the concrete surface or rubber mats [24]. Availability and use of dry, soft and deep bedding are essential for growth of calves [21,25]. Deep and dry bedding decreases heat loss in calves, thus preventing hypothermia [21]. The frequent addition of sufficient amounts of bedding in pens increases the use of lying posture that is closely related with sleeping and comfort in calves [26].

In Kenya, dairy farming is a significant contributor to the gross domestic product [27]. Dairy calves are raised to heifers for the replacement of cows in most smallholder farms. There is a need to facilitate not only optimal growth rate and performance but also comfort, rest, and welfare of calves. Improved animal welfare reduces the stress level in calves, enhances immune function and subsequently increases weight gain [5]. There have been several reports of calf growth, morbidity, and mortality on smallholder dairy farms in Kenya [28,29]. However, to the best of our knowledge, there has never been an assessment of calf comfort or its indicators on smallholder dairy farms in Kenya or other developing countries.

This study was aimed at describing calf comfort and determining the individual and pen level factors that affected calf comfort (in particular, calf leg hygiene) of smallholder dairy farms in Meru County, Kenya.

## Materials and Methods

### Ethical approval

The study was approved by the Research Ethics Board and the Animal Care Committee of the University of Prince Edward Island, the Naari Dairy Farmers Cooperative Society (NDFCS), and Farmers Helping Farmers (FHF), a partnering non-governmental organization with over 35 years of experience working with Kenyan farmers and farm groups.

### Informed consent

The study was explained orally to all participants, and signatures for informed consent were obtained from all the participants in the study.

### Study design and sampling method

This research was a cross-sectional study carried out in 38 dairy farms in the Naari region of Meru County in Kenya between May and August 2017. The full list of active farmers in the NDFCS was used as the initial sampling frame that was limited to 200 farms using simple random sampling. The following inclusion criteria were used to further identify 100 farms from the 200 farms for ongoing monitoring for the project: Zero-grazing units for the lactating cows and up to three cows per farm. Farms without calves and farms with calves older than 1 year were excluded from the study, leaving 52 calves, and 38 farms for the study.

### Data collection

The 52 calves were assessed for their: Biodata, welfare, health status, and management.

For biodata, the weight was measured in kg using a dairy cow heart girth weight tape. The height was measured using a height stick with a level that was placed at the withers. All calves were hybrids; thus, the breeds were described as cross-bred if the calves were visibly and predominantly Friesian, Guernsey, Ayrshire or Jersey, and indigenous if they were visibly and predominantly Zebu, Boran, or other local breeds. The body condition score was described using the 5-score chart that ranged from 1 (very thin) to 5 (excessively fat), with quarter-point increments [30].

For calf welfare data, the leg hygiene of the calves was assessed using a 5-score system [31] that included: (1) Very clean, (2) clean, (3) fair, (4) dirty, and (5) very dirty. Lameness observed in the calves was classified using a 3-point scoring system modified from a 5-point system [32] as absent (normal gait), mild (uneven gait), or severe (short striding gait with at least one limb or reluctance to put weight on at least one limb). Neck, carpal, and hock injuries were classified using a 3-score system: (1) No swelling and no hair loss, (2) minor swelling and/or bald area visible, and (3) medium/major swelling and/or bald area, broken skin or scab present developed by Cornell University and used in various studies [33,34].

For the calves kept in pens: Data on pen dimensions, floor type, and roof adequacy, type of bedding, pen hygiene, knee wetness, and knee impact test scores were collected. The pen dimensions were measured in cm. The knee impact test (from a crouched position on your feet, tipping forward, so your knees contact the floor surface) was used to determine how soft the stall surface was and was categorized into three possible levels: Normal, marginal, and hard. If the floor was soft and did not cause any level of discomfort on the knees, the floor was categorized as normal which indicated a passing grade on the knee impact test. If the floor was somewhat uncomfortable on the knees, such as a cement floor with a modest amount of bedding or a dirt floor that was compacted, then it was classified as marginal. If the floor caused extreme discomfort on the knees on impact, the floor was classified as hard and this indicated failure of the knee impact test.

The degree of wetness on the floor surface was assessed using the knee wetness test, which was categorized as normal if the knee was completely dry after about 10-15 s of knee contact on the floor, marginal if the knee had some noticeable moisture, and wet if the knee was completely wet after the contact with the floor. The knee wetness and impact tests have been used elsewhere to assess floor conditions for cattle [35]. We included a marginal category to the knee tests to adapt the tests to the highly variable stall management conditions that exist in Kenya where dirt (not sand) and crop waste are commonly used for floor surfaces.

The pen hygiene score reflected the leg hygiene score where the categories included: (1) Very clean, (2) clean, (3) fair, (4) dirty, and (5) very dirty. The adequacy of the roof (yes or no) was determined based on parameters such as the cover of the entire pen and the allowance of water to enter or not to enter into the pen through holes.

The health status of the calves was determined by conducting a physical examination of the calf that included but not limited to; heart rate, respiratory rate, color of mucous membranes, palpation of superficial lymph nodes, rumen movements (where applicable), skin condition, joints and feet examination, and examination of the umbilicus. Any abnormalities observed were recorded and treated on the farm level.

A questionnaire was administered to the 38 farmers face-to-face by the investigator using the native language of Kimeru to gather information on the housing management status of the pre-weaned and weaned calves.

### Data management and analysis

Collected data were entered manually into Microsoft Excel 2013 (Microsoft, Sacramento, California, USA), cleaned, coded, and imported to Stata^®^ 14.2 statistical software (StataCorp LLC, College Station, Texas, USA) for analysis. Descriptive statistics, such as means, standard deviations, and ranges, were determined for continuous variables, while proportions were calculated for binary and categorical variables.

Average daily weight gain (ADG) was estimated by dividing the difference between body weight during the study and estimated birth weight by the age of the calves in days. The average birth weight was estimated as an average from birth weights reported in smallholder farms in Africa [36-38]. Comparison of continuous variables across different groups was performed using the t-test, while comparison of categorical variables across different groups was performed using Chi-squared tests.

Univariable logistic regressions were used to determine unconditional associations of variables with calf cleanliness. This outcome of interest was generated by dichotomizing the leg hygiene score, with scores lower or equal to 2.5 categorized as clean, while scores >2.5 were categorized as dirty. This parameter was chosen for regression analyses because calf cleanliness represents an overall comfort parameter, and it had sufficient variability for regression analyses.

Variables with a p<0.2, on univariable analyses, were eligible to be fit in a multivariable logistic regression model to determine factors associated with calf dirtiness, while controlling for possible confounding effects. Since there were 52 calves in total but only 39 calves reared in pens, two multivariable models were used, one for each of the calf-level variables and one for the respective pen-level variables. Variables eligible for these separate models were also utilized to build a combined model of both calf-level and pen-level variables. These multivariable logistic regression models were fit through backward elimination, retaining variables with p<0.1 due to the small sample size. The final combined logistic regression model was assessed for two-way interactions, independence, linearity, goodness of fit, influential observations, and predictive ability.

## Results

The age of calves ranged from 1 week to 12 months with an average of 5.2±3.1 months, with more details provided in Table-1. Nearly half of the calves were heifers, while less than a quarter of calves were categorized as indigenous. The calves had a mean (and standard deviation) body condition score of 2.5±0.4, ranging from 1.5 to 3.5. Two calves showed signs of respiratory infection on physical examination that was characterized by coughing and abnormal lung sounds. The mean and standard deviation of body weight and height of the calves were 85±32.8 kg and 83.5±9.7 cm, respectively, while; the ADG was 0.50±0.45 kg.

**Table-1:** Calf and penlevel characteristics of calves in 38 smallholder dairy farms in Meru County, Kenya, 2017, along with the relative proportions andP** values associated with dirty leg hygiene scores in calves.

Factor	Groups	Frequency (%)	Percent dirty calves	p-value for differences between groups	Overall p-value

Calflevel factors (n=52 calves on 38 farms)					
Age (months)	<2.5	12 (23)	8.3 (1/12)		0.071
	≥2.5 and<5.5	14 (27)	50.0 (7/14)	0.041	
	≥5.5	26 (50)	38.5 (10/26)	0.081	
Breed	Cross-bred	44 (85)	38.6 (17/44)		n/a
	Indigenous	8 (15)	12.5 (1/8)	0.183	
Sex	Female	27 (52)	40.7 (11/27)		n/a
	Male	25 (48)	28.0 (7/25)	0.337	
BCS	>2.25(good)	37 (71)	24.3 (9/37)		n/a
	≤2.25 (poor)	15 (29)	60.0 (9/15)	0.018	
Health status	Healthy	50 (96)	34.0 (17/50)		n/a
	Non-healthy	2 (4)	50.0 (1/2)	0.646	
Housing	Indoors in pens	39 (75)	41.0 (16/39)		n/a
	Outdoors	13 (25)	15.4 (2/13)	0.108	

**Penlevel factors (n=39 calves in 35 pens on 28 farms)**					

Calf grouping	1 calf per pen	25 (64)	36.0 (9/25)		
	>1 calf per pen	14 (36)	50.0 (7/14)	0.396	n/a
Floor type	Dirt	29 (74)	31.0 (9/29)		n/a
	Concrete and wood	10 (26)	70.0 (7/10)	0.039	
Bedding type	None	15 (39)	33.3 (5/15)		0.034
	Sawdust and wood shavings	11 (28)	72.7 (8/11)	0.055	
	Crop waste	13 (33)	23.1 (3/13)	0.551	
Pen hygiene	Clean	9 (23)	33.3 (3/9)		0.091
	Fair	18 (46)	27.8 (5/18)	0.766	
	Dirty	12 (31)	66.7 (8/12)	0.138	
Knee impact	Normal	2 (5)	50.0 (1/2)		0.931
	Marginal	28 (72)	39.3 (11/28)	0.767	
	Hard	9 (23)	44.4 (4/9)	0.887	
Knee wetness	Normal	9 (23)	44.4 (4/9)		0.812
	Marginal	17 (44)	35.3 (6/17)	0.649	
	Wet	13 (33)	46.2 (6/13)	0.937	

p-value indicates significance of difference between proportions of dirty calves in the different categories of each variable and overall p-value indicates overall significance of the whole variable

Lameness, carpal lesions, and hock lesions were absent in all the examined calves, while a neck lesion was seen in one female 10 months old calf.

75% of the calves (39/52) were reared in pens on 28 separate farms (Table-1). The average pen area was 3.09±1.46 m^2^, with the number of calves per pen ranging from 1 to 3, with a mean of 1.4±0.5 calves per pen. The available pen area per calf averaged 2.52±1.56 m^2^, with a range of 1.02–7.64 m^2^. While the calves in the smallholder dairy farms were reared in housing systems that were quite different from each other across farms in the study, all the calf pens assessed had an appropriate roof. For the 39 calves kept in pens, 23% and 33% of them had a failed knee impact and knee wetness test, respectively, with 62% of pens having bedding and 26% of pen floors being wooden or concrete. The overall mean pen hygiene score was 2.9±0.9, while the mean leg hygiene score was 2.3±1.1. 65% (34/52) of all the calves observed were categorized as clean, with a leg hygiene score ≤2.5. For the 39 pens, 23 of them (59%) were categorized as clean.

Table-1 provides a summary of data on categorical calf- and pen-level factors, along with the relative proportions of calves with dirty leg hygiene scores.

Table-2 outlines the mean, standard deviation and range of pen and leg hygiene scores across three groups of bedding availability for 39 calves housed in pens. Leg hygiene scores and pen hygiene scores appear to be similar for the calves in pens with no bedding or bedded with sawdust or wood shavings. However, for the calves in pens with crop waste or other types of bedding, the pen hygiene scores are the dirtiest, while the calf hygiene scores are the cleanest. The differences in leg hygiene scores between categories of bedding were not significantly different. Availability of bedding, regardless of type, was associated with pen hygiene (χ^2^=7.90; p=0.019).

**Table-2:** Descriptive statistics of pen hygiene and leg hygiene scores across three types of bedding availability for 39 calves on 28 smallholder dairy farms in Meru County, Kenya, 2017.

Factor	Pen hygiene scores*			Leg hygiene scores*			n
		
Bedding	Mean±SD	Minimum	Maximum	Mean±SD	Minimum	Maximum	
None	2.53±0.72	1	4	2.27±1.12	1	4	15
Sawdust and wood shavings	2.95±1.17	1	4	3.05±1.42	1	5	11
Crop waste and others	3.15±0.80	1.5	5	2.00±0.79	1	3.5	13

* Pen and leg hygiene scores were assessed using a 5score system that included; 1 (very clean), 2 (clean), 3 (fair), 4 (dirty), and 5 (very dirty). SD=Standard deviation

On univariable analyses, variables with a p<0.2 included: Age, breed, BCS, and the housing variable, all which were eligible to be fit into the preliminary multivariable logistic regression model of calf-related variables associated with calf cleanliness among the 52 calves. Type of floor, type of bedding and pen hygiene score were variables with a p<0.2 on univariable analyses, and therefore eligible to be fit into the preliminary multivariable logistic regression model of pen-level variables associated with calf cleanliness among the 39 calves in pens.

For the multivariable logistic regression of calf-related variables, body condition score had a slight correlation with age (r=0.20). The probability of calves being categorized as dirty was higher in older calves (2.5–5.5 months) in comparison to younger calves (odds ratio [OR]=36.8, p=0.008). Cross-bred calves had higher odds of having a dirty score compared to indigenous calves (OR=22.6, p=0.021), while indoor-reared calves were dirtier than outdoor-reared calves (OR=8.4, p=0.028). Table-3 illustrates these results.

**Table-3:** Calflevel factors associated with leg dirtiness of 52 calves on 38 smallholder dairy farms in Kenya, 2017.

Factor	OR	95% CI	p-value
Age of calves			
≤2.5 months	Reference	Reference	0.031*
>2.5 and≤5.5 months	36.7	[2.51, 538.95]	0.008
>5.5 months	8.5	[0.88, 81.84]	0.064
Breed			
Indigenous	Reference	Reference
Crossbred	22.6	[1.58, 321.38]	0.021
Type of housing			
Outdoors	Reference	Reference
In pens	8.4	[1.25, 56.05]	0.028

* Overallp-value

The multivariable logistic regression model of pen-related variables indicated that the odds of calves being dirty were 6.8 times higher in calves kept in groups compared to the odds of calves housed individually (p=0.097). Calves in pens categorized as dirty had 6.4 times higher odds of being categorized as dirty (p=0.088) compared to calves in pens categorized as clean. The odds of calves having a dirty score (>2.5) were higher in pens with bedding than calves in pens without bedding (p=0.025). In addition, use of sawdust or wood shavings as bedding increased the odds of calves being categorized as dirty by 13.2 times versus no bedding at all (p=0.017).

When combining the calf- and pen-related factors in the multivariable logistic regression model for calf leg hygiene in the 39 calves raised in pens, concrete or wood floors, poor body condition and use of bedding were risk factors associated with calves categorized as dirty (Table-4). There were no interactions between the main effects of the final model. Test fitness indicated that the final logistic regression model was appropriate (χ^2^=3.34; p=0.503).

**Table-4:** Final multivariable logistic regression model of calf and penlevel factors associated with dirtiness of 39 calves on 28 smallholder dairy farms in Kenya in 2017.

Factor	OR	95% CI	p-value
Body condition score			
>2.25 (good)	Reference	Reference
≤2.25 (poor)	17.06	[1.567, 185.781]	0.020
Floor type			
Dirt	Reference	Reference
Wood or concrete	7.91	[1.025, 61.131]	0.047
Bedding type			
None	Reference	Reference
Sawdust, wood shavings or crop waste	12.55	[1.043, 150.865]	0.046

OR=Odds ratio, CI=Confidence interval

## Discussion

This is the first assessment of calf comfort among smallholder dairy farms in tropical countries and provides useful information for guiding extension officers and animal health professionals on how to assess basic parameters of calf welfare, without impairing the 5 domains of welfare recently proposed [39]. This study demonstrated that the examined population of calves had reasonable space allowance, but one-quarter to one-third of the indoor-reared calves were housed in pens with suboptimal flooring and bedding management, leading to failed knee impact and knee wetness tests, dirtier pens, and therefore dirtier calves (Table-1). Calves with leg hygiene scores categorized as dirty were significantly associated with wood or concrete floors and bedding use that was not properly managed (Table-3).

There is limited information on calf- and pen-related factors associated with calf comfort in smallholder dairy farms in Kenya. However, leg hygiene scores of the calf can be used as indicators of calf comfort.

In the calf-related multivariable logistic regression model, indoor calves (75%) housed in pens were dirtier than outdoor-reared calves, and this could be a result of poor management practices, such as delays in dirty bedding and/or manure removal. The 13 calves kept outside were either tethered on a long rope or grazing in a field, and therefore their frequent movement during the day, and the availability of more space for lying, standing and movement explain the low probability of having dirty leg hygiene scores (OR=0.12) in comparison to indoor calves. A survey of calf management practices in Quebec, Canada showed that inappropriate calf housing systems such as tie-stalls (13.9%) and attachment against a wall (5.7%) were risk factors for poor calf welfare [6]. The outdoor reared calves were not included in the multivariable regression analyses of combined calf- and pen-level factors.

The average lying space of 2.5 m^2^ available for each calf in our study was higher than that recommended (1.4 m^2^) for calves with an average weight of up to 160 kg in Germany [3]. Calves housed in groups (36%) were categorized as dirtier (Table-1) than individually housed calves (64%), a fact that could be explained by limited space availability per calf (group calves had 1.5±0.5 m^2^, while individual calves had 3.1±1.7 m^2^), in addition to poor manure and bedding management practices in group-housed calves.

A higher body condition score can be attributed to sufficient feed intake, good health status, and subsequently larger body weight which was observed in calves with body condition scores >2.5 (p=0.019). Calves with higher body condition scores (>2.25) were also less likely to have dirty leg scores which could be due to better management practices by the farmers, such as the regular removal of manure. A recent study stipulated that changes in an animal’s BCS may reflect the adjustment of its welfare status, but this relationship is also affected by other factors [40].

The estimated average daily gain (0.5 kg) was higher than the mean daily gain reported in a study carried out in the Nakuru region of Kenya [41], and this difference may be due to the larger sample size used in the previous study (n=601), and the measurement of the birth weight of calves compared to the present study, where the sample size was small (n=52) and the birth weight was an estimate from three different studies in Africa. Cross-bred calves had a higher mean body weight (p=0.029) and average daily gain (p=0.015), which could be attributed to their faster growth rate in comparison to the indigenous-bred calves [42]. Similar results were reported in a study implemented with Sahiwal Cattle in India [43]. The higher probability of having dirty legs was observed in older calves (>2.5 months), and it could be a result of the larger amounts of fecal excrement in pens of these calves compared to the young ones regardless of the lying space available per calf.

In the pen-related (and combined) multivariable logistic regression model, calves kept in pens with wooden or concrete floors had significantly higher odds of being dirty (Table-4) which could be due to poor drainage of urine or wet manure or delayed cleaning of the pen.

Availability of any bedding in the pens increased the odds of a dirty leg hygiene score of the calves which could be due to the delayed removal of dirty wet bedding and the insufficient or delayed addition of new bedding in the pens. The higher odds of calves being dirty in pens bedded with sawdust and wood shavings could be due to low availability and the high cost of acquiring new sawdust and wood shavings in comparison to crop waste that is readily available at the farms. Other studies have shown that the type of bedding used in rearing calves can have an effect on calf cleanliness [20].

As expected, calf cleanliness was strongly related to the pen hygiene, since a dirty score for the pen was closely related with a large portion of the pen being wet, a fact that increases the likelihood of the calves being dirty. We also speculate that poor moisture absorbency of crop waste may explain the lower pen hygiene level in crop-waste bedded pens compared to sawdust or wood shavings bedded pens (Table-2). Crop waste used as bedding may imply that new bedding is added as frequently as the cow is fed new pasture, which could explain the lower leg hygiene scores of calves in crop-waste bedded stalls, although the pen hygiene scores were the highest (Table-2).

## Conclusion and Recommendations

Overall, some welfare aspects of the examined calves were adequate, with nearly adequate pen sizes, sufficient roofs, and knee impact, and knee wetness tests. Moreover, 65% of calves had relatively high leg hygiene levels. However, there was room for improvement, particularly in overall pen hygiene where 69% of the pens were categorized as dirty. Rearing calves in pens with sawdust and wood shavings were negatively associated with the calf leg hygiene, providing a reminder that the addition of clean, dry bedding should be accompanied with removal of the previous wet bedding. The cleanliness of the calves was also associated with the type of floor in the pens, being worse in concrete or wooden floors, likely because they cannot absorb moisture. If possible, calves should be reared on surfaces that allow moisture to drain away, such as sand or sandy dirt, which makes it easier to keep calves dry with less labor (daily manure removal and clean, dry bedding addition). If calves are reared on wood or concrete floors, they require manure removal and dry bedding addition/management at least daily, if not twice daily. These recommendations would ensure that calves would be kept in pens with clean, soft, and dry bedding to optimize their performance. Farmers should be trained on the importance of good housing management practices of the calves to enhance their growth and welfare. Further, research should be carried out to determine the solid feed intake, growth rate and diseases of dairy calves in Kenya.

The body condition score could be a good indicator of good welfare of the calves since it was associated with leg hygiene and is an indicator of nutritional management. Further, research should be carried out to determine relationships between calf welfare parameters, body condition score, feed intake, growth rate, and diseases of dairy calves in Kenya.

The small sample size for calf-related factors (52) and pen-related factors (39) limited the number of significant relationships and associations observed. The small number of farms (38) also may have limited the variation in calf cleanliness. Furthermore, the cross-sectional nature of this study provides us with data on housing, calf comfort, and hygiene, however without information on previous housing, welfare, and hygiene levels. Other calf welfare traits, such as the five domains of welfare, and detailed calf management practices (including feeding) would also be useful parameters to measure. A larger cohort study of more calves on more farms measuring the 5 domains of welfare would add perspective to the observations in our study.

## Authors’ Contributions

EKK, JVL, GKG, and SLM were involved in the study design, data analysis, and writing of the manuscript. All authors read and approved the final manuscript.
